# Splenic and Pancreatic Abscess Secondary to Chronic Corticosteroid Use: A Case Report and Literature Review

**DOI:** 10.7759/cureus.84325

**Published:** 2025-05-18

**Authors:** Fernando Sánchez Martínez, Oscar Sebastian Salinas Rosas, Carlos Ronaldo Martínez Mateo, Alejandro Aguilar Sabori, Erik Ponce Graciano, David Alejandro Rodríguez Herrera, María Fernanda Vázquez Páez, Angeles Yasunari Cortes Garcia

**Affiliations:** 1 Surgery, Clinica Hospital Constitución, Instituto de Seguridad y Servicios Sociales (ISSTE), Monterrey, MEX; 2 Surgery, Universidad Autónoma del Estado de Morelos, Cuernavaca, MEX; 3 Surgery, Universidad Nacional Autónoma de México, Mexico City, MEX; 4 Surgery, Universidad Autónoma de Baja California, Tijuana, MEX; 5 Surgery, Hospital General del Sur Eduardo Vázquez Navarro, Puebla, MEX; 6 Surgery, Unidad Medica de Alta Especialidad No. 71, Instituto Mexicano Del Seguro Social, Torreón, MEX; 7 Surgery, Unidad Medica de Alta Especialidad, Instituto Mexicano Del Seguro Social Puebla, Puebla, MEX; 8 Surgery, Hospital Regional de Alta Especialidad, Instituto de Seguridad y Servicios Sociales (ISSTE), Morelia, MEX

**Keywords:** corticosteroids, immunosupression, management of splenic abscess, open distal pancreatectomy, total splenectomy

## Abstract

Splenic abscess is a rare but potentially life-threatening condition often associated with immunosuppressive states. We present the case of a 62-year-old man with a history of chronic corticosteroid use who developed fever, anorexia, weight loss, and left upper quadrant abdominal pain. Laboratory studies revealed leukocytosis and elevated inflammatory markers. Contrast-enhanced computed tomography identified multiloculated abscesses in the spleen and pancreatic tail. The patient underwent exploratory laparotomy, splenectomy, and distal pancreatectomy, with a favorable postoperative course complicated only by a superficial surgical site infection. Splenic abscess typically results from hematogenous spread, and its diagnosis is often delayed due to nonspecific symptoms. Imaging, particularly contrast-enhanced CT, is critical for early detection. Management strategies include percutaneous drainage or surgical intervention, depending on the patient's clinical status. Although corticosteroid-induced immunosuppression is a rare risk factor, it should be recognized as a potential cause. Early diagnosis, source control, and targeted antibiotic therapy are crucial for optimizing patient outcomes.

## Introduction

Splenic abscess is a rare but serious medical condition, historically associated with high mortality rates. Autopsy studies report an incidence between 0.2% and 0.7% [1, 2], and although advances in diagnosis and management have improved outcomes, mortality can still reach up to 6-40% [1-4]. Typically, the condition presents with a classic triad of fever, left upper quadrant abdominal pain, and a palpable mass, yet many patients exhibit nonspecific symptoms, leading to delayed diagnosis [5-7].

Risk factors include immunosuppressive states, notably diabetes mellitus, HIV infection, malignancy, or, as highlighted in this report, chronic corticosteroid use [1, 7]. Imaging, particularly contrast-enhanced computed tomography (CT), is crucial for early diagnosis, revealing irregular cystic lesions with debris, air, and fluid [8, 9].

Despite a lack of randomized controlled trials comparing management strategies, current practice emphasizes early source control via percutaneous drainage or splenectomy, supported by prolonged antibiotic therapy [1, 6, 10, 11]. We present a case of splenic and pancreatic abscess successfully managed with splenectomy and distal pancreatectomy in the context of chronic corticosteroid use.

## Case presentation

A 62-year-old man with a history of chronic corticosteroid and herbal remedy use for joint pain for eight months presented with a one-month history of anorexia, weight loss, fever, and left upper quadrant abdominal pain. He denied intravenous drug use and other significant symptoms.

Initial laboratory findings showed hemoglobin 10.8 g/dL, hematocrit 33.9%, leukocytes 16.56 ×10⁹/L, platelets 465 ×10⁹/L, neutrophils 9.51 ×10⁹/L, glucose 87.6 mg/dL, blood urea nitrogen 31.54 mg/dL, urea 67.5 mg/dL, creatinine 2.31 mg/dL, sodium 135 mmol/L, potassium 5.19 mmol/L, chloride 103.4 mmol/L, C-reactive protein 9.96 mg/L, and procalcitonin 0.30 ng/mL.

**Figure 1:**
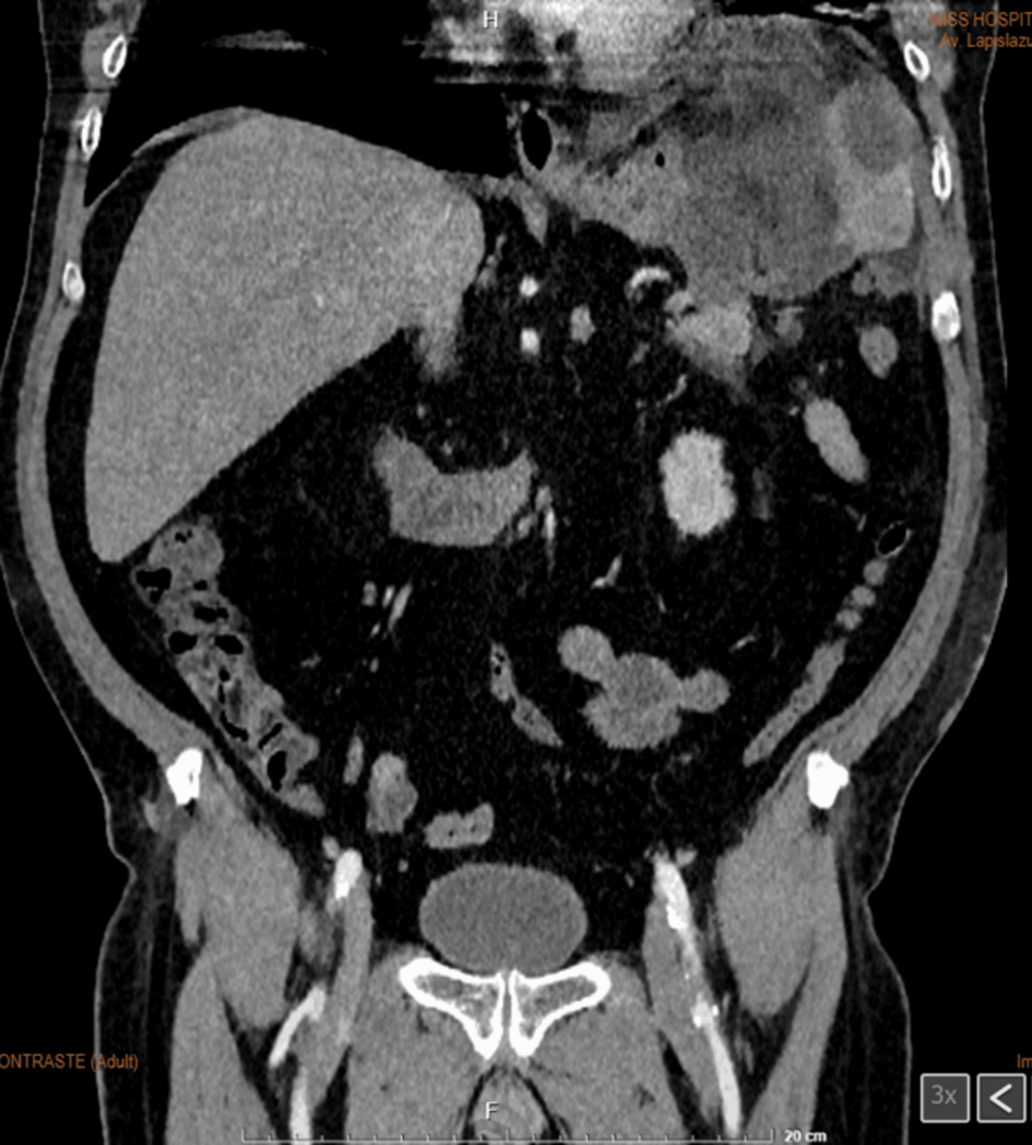
Contrast-enhanced abdominal computed tomography in coronal view showing a collection localized within the spleen with central density variation.

**Figure 2:**
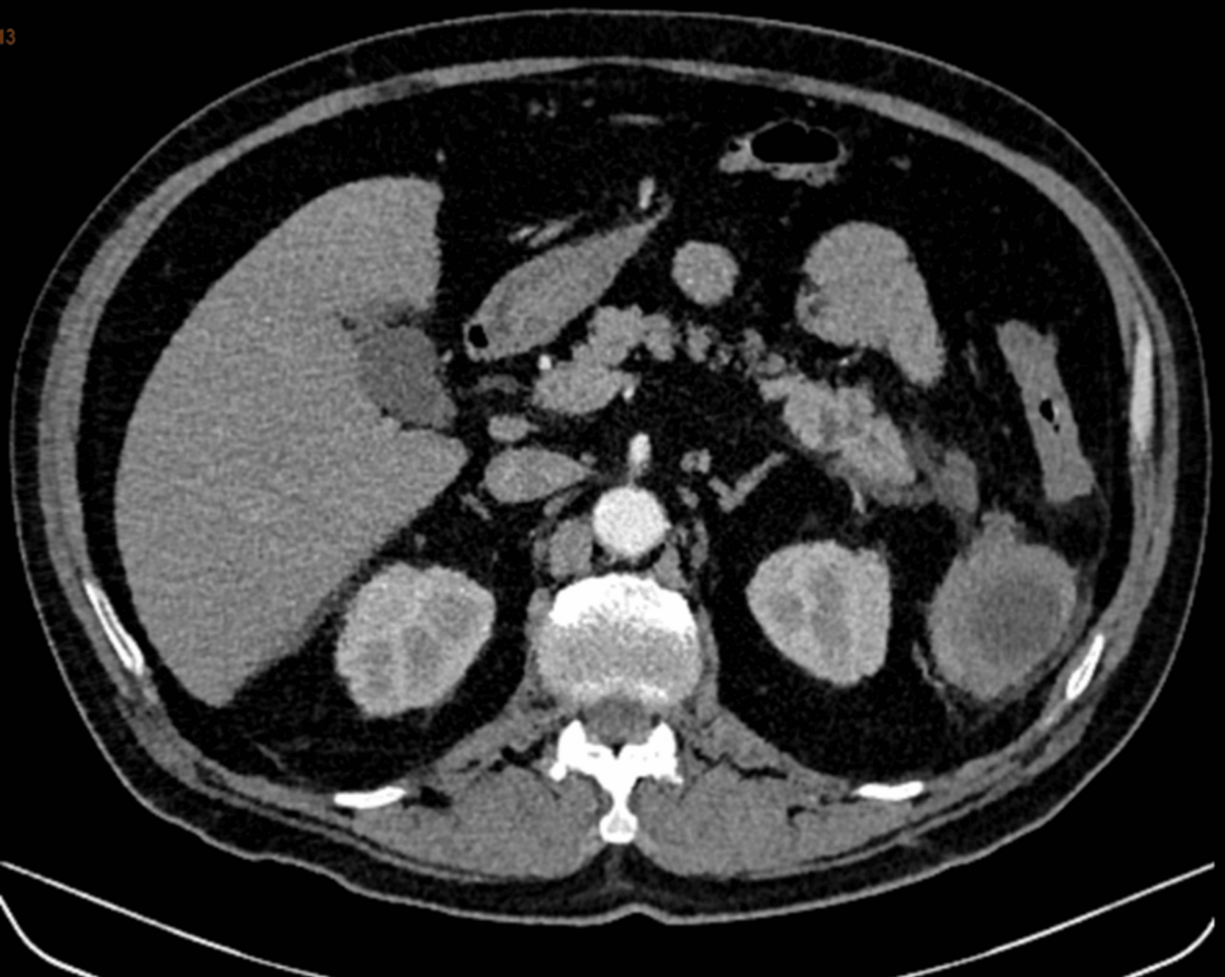
Axial view of spleen showing peripheral enhancement with two intraparenchymal collections.

**Figure 3:**
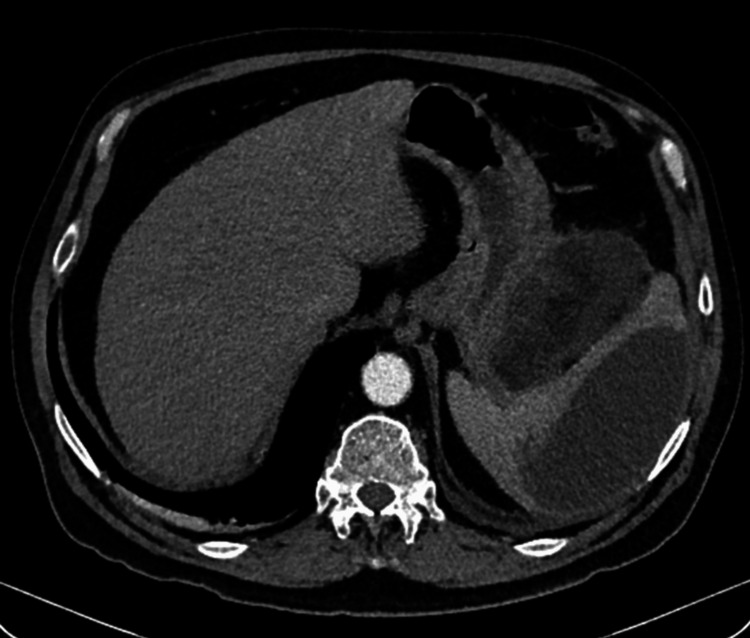
Axial view of the tail of the pancreas and lower pole of the spleen showing the presence of abscesses.

A contrast-enhanced abdominal CT scan revealed a multiloculated lesion in the spleen with associated involvement of the pancreatic tail (Figures 1-3).

**Figure 4:**
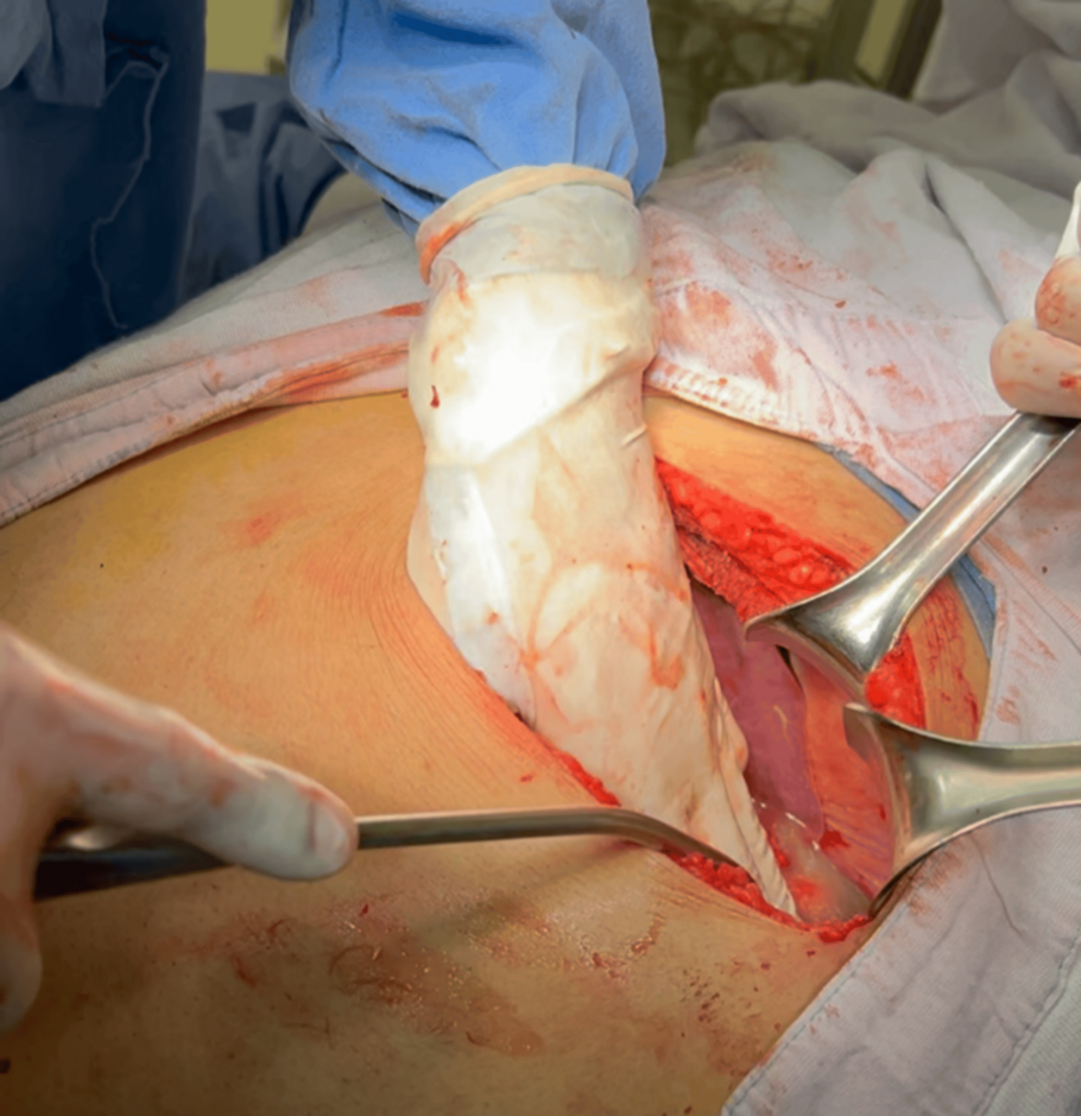
Exploratory laparotomy through a left subcostal incision with purulent material drainage upon manipulation.

**Figure 5:**
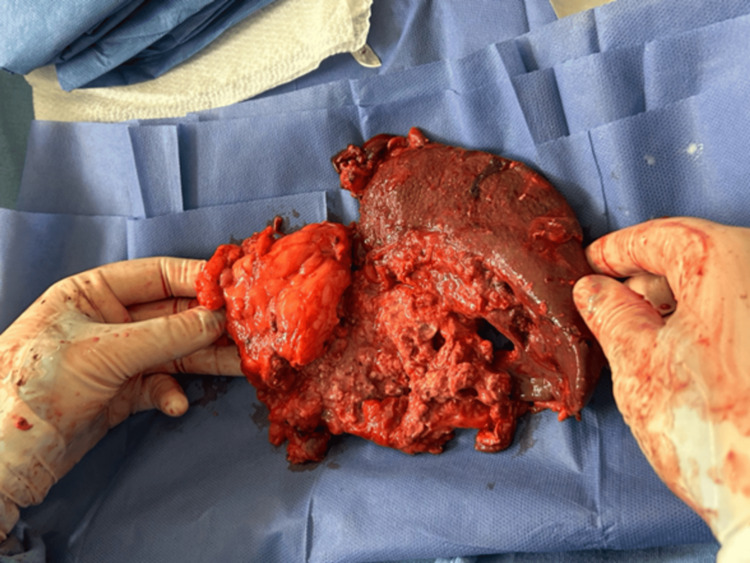
Resected specimen from splenectomy and distal pancreatectomy showing macroscopic alterations of splenic and pancreatic tissue corresponding to abscess sites.

Empiric antibiotic therapy with ceftriaxone, metronidazole, and meropenem was initiated. Surgical intervention via a left subcostal incision was performed, consisting of splenectomy and distal pancreatectomy (Figure 1). Postoperative recovery was favorable (Figures 4,5), though the patient developed a superficial surgical site infection, prolonging hospitalization to 25 days. Blood cultures and abscess fluid cultures were negative, likely due to suboptimal culture techniques secondary to low clinical suspicion of atypical organisms.

## Discussion

Epidemiology and risk factors

Splenic abscesses are uncommon, predominantly affecting men with a mean age of 57 years [1]. Immunosuppressive conditions, primarily diabetes mellitus, are the most frequent predisposing factors [1,7,11], but chronic corticosteroid use, as in this case, is a less common but recognized risk (approximately 1% of etiologies) [1].

The spleen serves as a critical filter for bloodstream pathogens, and hematogenous spread remains the principal pathophysiological mechanism [7,9]. Alternative mechanisms include secondary infection of infarcted splenic tissue or contiguous spread from intra-abdominal infections.

Clinical presentation

While the classic triad of fever, left upper quadrant pain, and a palpable mass is often cited, it is inconsistently observed, with many patients presenting only with fever or vague abdominal discomfort [5-7]. Fever is the most consistent clinical sign and should prompt imaging evaluation in at-risk patients.

Diagnostic imaging

Contrast-enhanced CT is the diagnostic modality of choice, with typical findings including irregular, cystic lesions containing debris, air, and fluid [8,9]. Fungal and mycobacterial abscesses tend to be multifocal, whereas bacterial abscesses may be unilocular or multilocular [12]. Interestingly, acute abscesses may not enhance on imaging, particularly in immunocompromised hosts, possibly due to impaired inflammatory response [8,9].

In this case, CT imaging enabled early diagnosis, guiding prompt surgical intervention.

Microbiological considerations

Blood cultures are positive in approximately 71.85% of cases, and abscess fluid cultures in up to 93.5% [6]. The most commonly isolated organisms include *Escherichia coli* and *Enterococcus* spp. [1,6]. Notably, in certain populations, *Mycobacterium tuberculosis *and *Candida* spp. are emerging pathogens [11].

Despite negative cultures in this patient, it is suspected that inadequate culturing techniques, particularly a lack of fungal and mycobacterial media, contributed to the absence of pathogen identification.

Management strategies

There is no consensus regarding the optimal treatment of splenic abscesses, due to the absence of comparative clinical trials. Nevertheless, source control-whether via percutaneous drainage or surgical splenectomy-remains the cornerstone of therapy.

Percutaneous drainage is often attempted first in accessible collections, particularly in patients unfit for surgery. Splenectomy is preferred when drainage is not feasible or when complicated by multifocal disease, hemorrhage, or associated pancreatic involvement, as in our case. Antibiotic therapy duration is individualized based on clinical response, with an average treatment course of 45 days. The "STOP-IT" trial suggested that shorter antibiotic regimens may be appropriate once effective source control is achieved [10]. Importantly, medical therapy alone has been reported as successful in up to 75% of selected patients [1,6], underscoring the need for a patient-tailored approach.

## Conclusions

Splenic abscess remains a rare but life-threatening condition, particularly in immunosuppressed patients. Early diagnosis through clinical suspicion and appropriate imaging is essential. Management should prioritize source control, either percutaneously or surgically, complemented by tailored antibiotic therapy. Our case highlights the successful surgical treatment of a splenic and pancreatic abscess secondary to chronic corticosteroid use and reinforces the importance of recognizing atypical risk factors to optimize patient outcomes.
